# High expression of Zinc-finger protein X-linked promotes tumor growth and predicts a poor outcome for stage II/III colorectal cancer patients

**DOI:** 10.18632/oncotarget.7547

**Published:** 2016-02-21

**Authors:** Xuebing Yan, Zezhi Shan, Leilei Yan, Qingchao Zhu, Liguo Liu, Bing Xu, Sihong Liu, Zhiming Jin, Yuping Gao

**Affiliations:** ^1^ Department of General Surgery, The Sixth People's Hospital Affiliated to Shanghai Jiao Tong University, Shanghai, China; ^2^ Department of Medicine, Soochow University, Suzhou, Jiangsu Province, China; ^3^ Center for Reproductive Medicine, Xinhua Hospital, School of Medicine, Shanghai Jiao Tong University, Shanghai, China

**Keywords:** colorectal cancer, ZFX, tumor growth, prognosis, DUSP5

## Abstract

Zinc-finger protein X-linked (ZFX) was recently identified as a novel oncoprotein in several human malignancies. In this study, we examined the correlation between ZFX expression and the clinical characteristics of stage II/III CRC patients, as well as the molecular mechanism by which ZFX apparently contributes to CRC tumor progression. Using immunohistochemistry, we detected expression of ZFX in CRC tissues collected from stage II/III patients and determined that its expression correlated with tumor differentiation and stage. Survival analysis indicated that patients with high ZFX expression had poorer overall and disease-free survival. ZFX knockdown in SW620 and SW480 CRC cells significantly inhibited cell proliferation and colony formation, enhanced apoptosis and induced cell cycle arrest. It also enhanced the sensitivity of CRC cells to 5-Fu. In a xenograft model, ZFX knockdown suppressed *in vivo* CRC tumor growth. Microarray analysis revealed the primary target of ZFX to be DUSP5. Whereas ZFX knockdown increased DUSP5 expression, DUSP5 knockdown rescued ZFX-mediated cell proliferation in ZFX knockdown cells. These findings demonstrate that ZFX promotes CRC progression by suppressing DUSP5 expression and suggest that ZFX is a novel prognostic biomarker and potentially useful therapeutic target in stage II/III CRC patients.

## INTRODUCTION

Colorectal cancer (CRC) is the third most commonly diagnosed cancer in males and second in females worldwide [1]. In the United States, it is the third leading cause of cancer-related mortality, accounting for an estimated 50,310 deaths in 2014 [2]. Although in recent years there has been a substantial reduction in the incidence and mortality of CRC, thanks to population-based screening and removal of high-risk adenomas [3, 4], the 5-year survival rate after surgical treatment continues to be poor among a considerable fraction of CRC patients [5]. Furthermore, due to high disease heterogeneity, CRC patients at different stages experience varied outcomes and respond differently to the same therapeutic strategy, resulting in inevitable under- or over-treatment. This clinical problem appears to be more prominent in patients with stage II/III cancer, who could potentially be cured by radical surgery combined with tailored chemotherapy [6, 7]. It is well established that the malignant progression of CRC is a consequence of complex interactions among numerous genetic and epigenetic factors. Therefore, identification of novel molecular biomarkers, which can predict the response of antitumor therapy, could be potentially useful for prognosis assessment and therapy determination in CRC patients.

Zinc-finger protein X-linked (ZFX) is a member of Krüppel-type zinc finger protein family and was originally identified as a critical regulator of self-renewal in both embryonic and hematopoietic stem cells [8]. It was recently suggested that ZFX is a novel oncoprotein frequently overexpressed in human malignances, including breast, renal and hepatocellular carcinoma [9–11]. In addition, ZFX targeting has been shown to effectively inhibit the growth of gastric cancer cells *in vitro* and *in vivo* [12]. Increased expression of ZFX negatively correlates with microRNA-144 expression and may contribute to bone marrow metastasis in gastric cancer [13]. In an earlier study, we showed that high expression of ZFX is frequently detected in CRC tissues and that it may predict poor overall survival in those patients [14]. Whether ZFX expression is any more useful for predicting patient outcome or a therapeutic response than current prognostic indicators, like TNM staging, remains unknown, however. Moreover, the molecular mechanism by which ZFX apparently contributes to CRC progression has not yet been studied. In the present study, therefore, we assessed the clinical significance of ZFX expression in a cohort of 290 stage II/II CRC patients, focusing on the role of ZFX in CRC progression and the molecular mechanism underlying its action.

## RESULTS

### Correlation of ZFX expression with the clinical characteristics of stage II and III CRC patients

**Figure 1:**
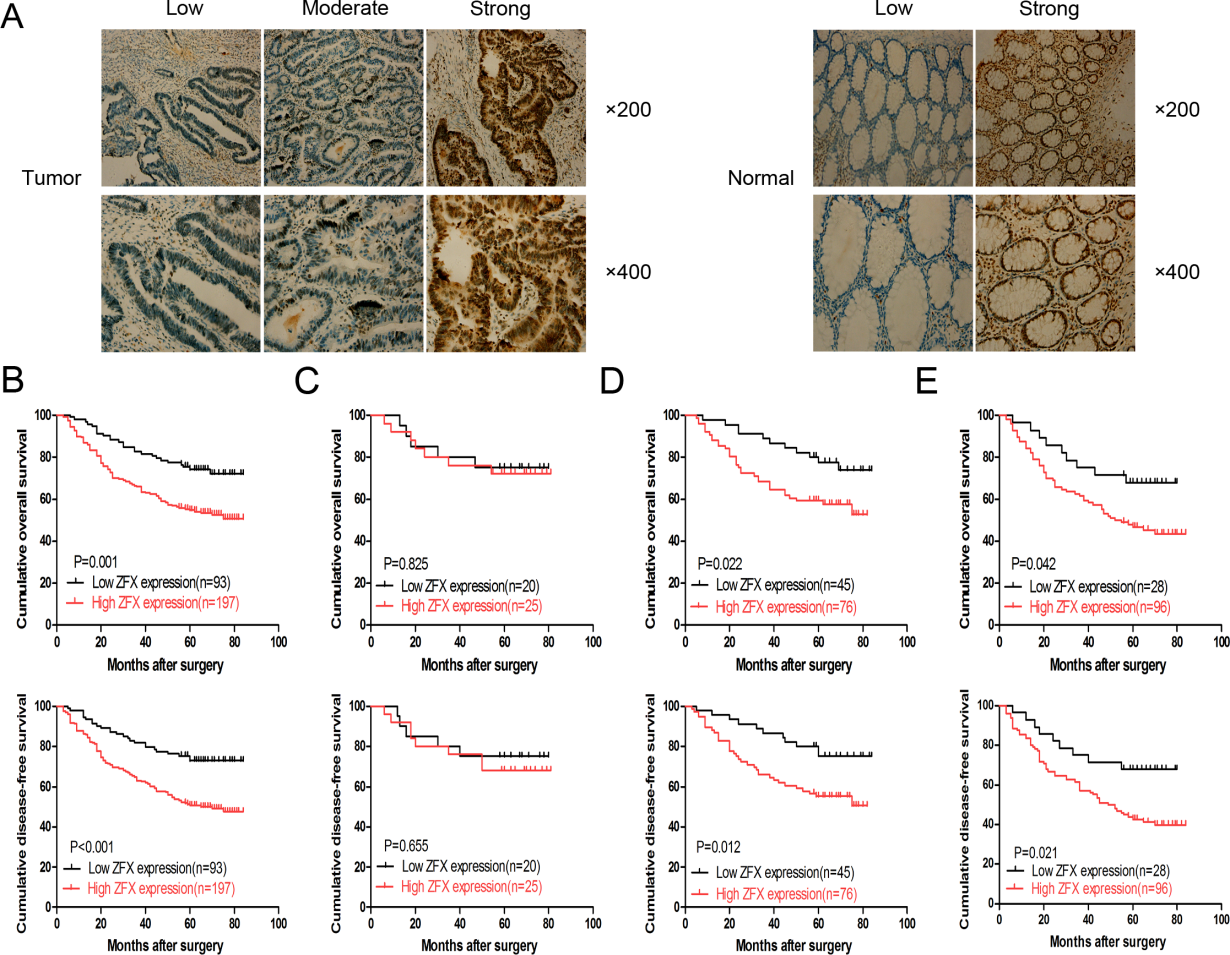
Expression and clinical significance of ZFX in colorectal cancer (CRC) (**A**) Representative immunohistochemical staining of ZFX in CRC tissues (left) and their matched normal tissues (right) at 200× and 400× magnification. (**B**) Overall survival (OS) and disease-free survival (DFS) curves for a cohort of stage II/III CRC patients. (**C**) OS and DFS curves for stage II CRC patients who received no chemotherapy. (**D**) OS and DFS curves for stage II CRC patients who received chemotherapy. (**E**) OS and DFS curves of stage III CRC patients who received chemotherapy.

Based on immunohistochemical staining analysis, ZFX expression was predominantly observed in the nucleus of tumor cells from CRC samples, as shown in the left panel of Figure 1A. The right panel of Figure 1A shows the ZFX expression in the normal matched control samples. A total of 197 CRC tissue samples from 290 (67.9%) patients analyzed exhibited high ZFX protein expression. In addition, ZFX expression correlated with tumor differentiation (*p* = 0.005) and TNM stage (*p* = 0.003), while no correlation was observed with other clinical parameters, including gender (*p* = 1.000), age (*p* = 0.900), tumor location (*p* = 0.706) and tumor size (*p* = 0.799) (Table 1).

**Table 1:** Correlation between ZFX expression and CRC clinicopathological characteristics

Characteristics	Total	ZFX expression		*P* value
		Low	High	
**Gender**				
Male	171	55	116	1.000
Female	119	38	81	
**Age**				
≤ 60	126	41	85	0.900
> 60	164	52	112	
**Tumor location**				
Colon	135	45	90	0.706
Rectal	155	48	107	
**Tumor differentiation**				
Well/moderate	192	72	120	0.005
Poor	98	21	77	
**Tumor size**				
≤ 5 cm	167	55	112	0.799
> 5 cm	123	38	85	
**TNM stage**				
II	166	65	101	0.003
III	124	28	96	

ZFX expression was also analyzed for its correlation with overall survival (OS) and disease free survival (DFS) among CRC patients. Following stratification of the patients based on ZFX expression, a complete cohort with higher ZFX expression was observed to have statistically lower OS (*p* = 0.001) and DFS (*p* < 0.001) rates than a cohort with lower ZFX expression (Figure 1B). Further analysis of the patients, stratified based on postoperative chemotherapy, also revealed a positive correlation between ZFX expression and low OS and DFS rates. This was not observed exclusively in stage II patients, but also occurred in stage III patients (Figure 1D and 1E, respectively). However, for stage II patients treated without postoperative chemotherapy, there was no significant correlation between ZFX expression and OS (*p* = 0.825) or DFS (*p* = 0.655) (Figure 1C). Univariate analysis indicated that ZFX expression, tumor differentiation and TNM stage were all prognostic factors for OS in stage II and III patients (*p* = 0.001, *p* = 0.019, and *p* = 0.005), while multivariate analysis confirmed that only ZFX expression and TNM stage were independent prognostic factors for OS in stage II and III patients (*p* = 0.007 and *p* = 0.034) (Table 2).

**Table 2:** Univariate and multivariate analysis for prognostic factors in stage II/III CRC patients

Variables	Univariate analysis			Multivariate analysis				
	RR	95% CI	*P* value	RR	95% CI	*P* value
Gender	0.930	0.643–1.344	0.697			
Age	1.147	0.794–1.656	0.464			
Tumor location	1.082	0.753–1.555	0.671			
Tumor size	0.863	0.597–1.248	0.433			
Tumor differentiation	1.553	1.076–2.241	0.019	1.383	0.954–2.005	0.087
TNM stage	1.688	1.176–2.423	0.005	1.488	1.031–2.150	0.034
ZFX expression	2.085	1.341–3.244	0.001	1.858	1.185–2.912	0.007

### Lentivirus-mediated ZFX knockdown in CRC cells

**Figure 2:**
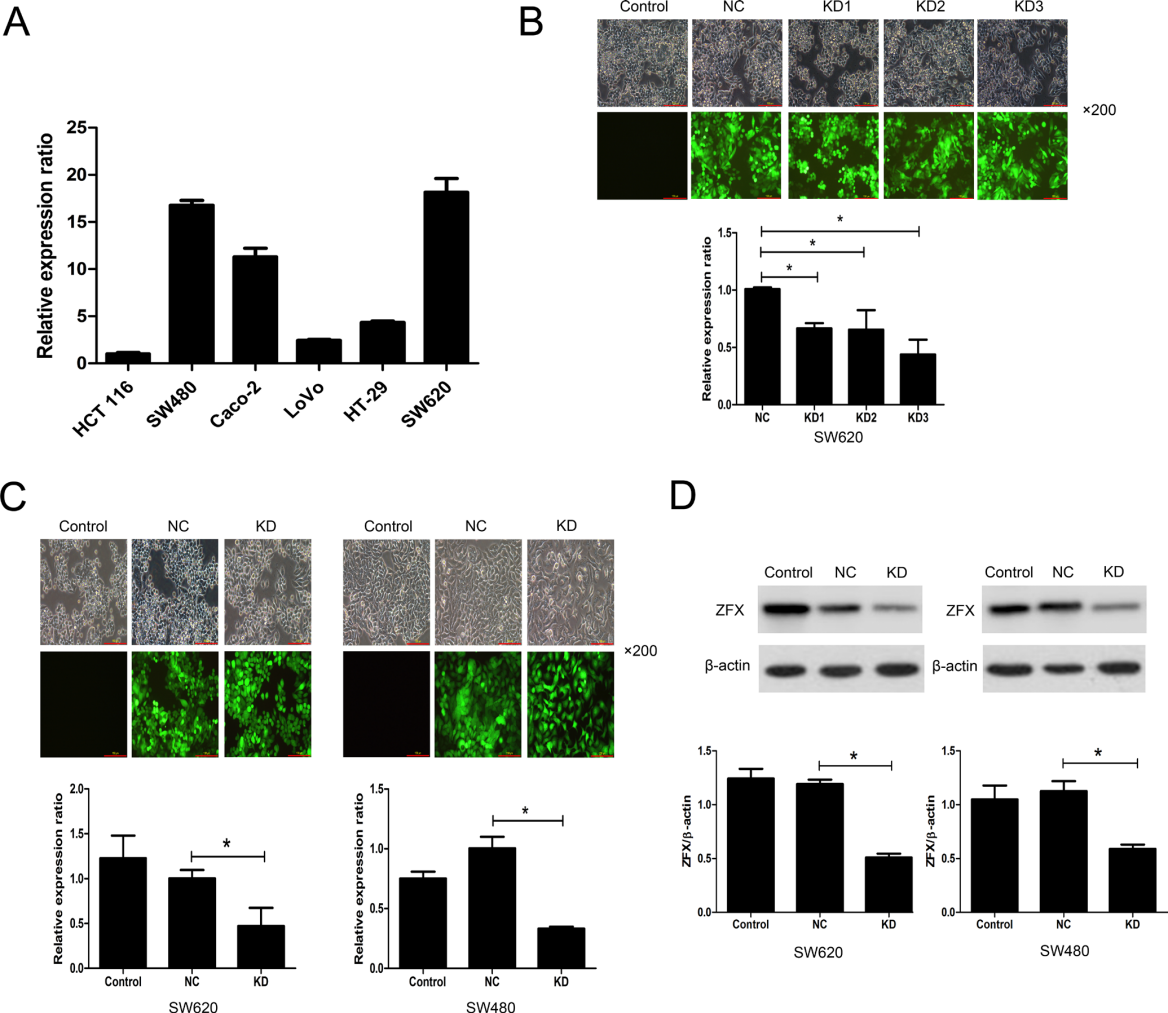
Analysis of ZFX expression and its lentivirus mediated shRNA knockdown (**A**) RT-PCR analysis of ZFX mRNA expression in two CRC cell lines. (**B**) The transfection and knockdown efficiency of each lentivirus shRNA (KD1, KD2 and KD3) in SW620 cells as evaluated using fluorescence microscopy and RT-PCR (bottom panel), respectively. (**C**) RT-PCR analysis confirming the transfection and knockdown efficiencies (bottom panel) of KD3 lentivirus shRNA in SW620 and SW480 cells. (**D**) Western blots confirming the knockdown efficiency of KD3 shRNA in SW620 and SW480 cells. *P < 0.05.

ZFX expression was initially analyzed in several CRC cell lines using RT-PCR. Two cell lines, SW620 and SW480, abundantly expressed ZFX, exhibiting relative expression levels of 18.16 ± 1.45 and 16.76 ± 0.51, respectively (Figure 2A). SW620 and SW480 cells were therefore selected for subsequent analysis of the functional role of ZFX in CRC. To assess their ability to ablate ZFX expression in these cells, three different shRNAs (KD1, KD2 and KD3) cloned into GFP-expressing lentiviral vectors were screened in SW620 cells. Based on the GFP expression, the lentiviral infection efficiency appeared very similar for all three shRNAs (Figure 2B, top panel). Moreover, RT-PCR analysis showed that ZFX mRNA expression was significantly downregulated by all shRNAs as compared to the negative control (NC) shRNA (NC vs. KD1: *p* = 0.0003; NC vs. KD2: *p* = 0.022; NC vs. KD3: *p* = 0.0016) (Figure 2B, bottom panel). Among the analyzed shRNAs, KD3 appeared to have the greatest mean knockdown efficiency (56.69%), and was therefore selected for subsequent experiments. The knockdown efficiency of KD3 in both SW620 and SW480 cells were also confirmed by RT-PCR (Figure 2C) and western blot (Figure 2D) before proceeding with further *in vitro* experiments (all *p* < 0.05).

### Effect of ZFX knockdown on CRC cell proliferation, apoptosis and cell cycle

**Figure 3:**
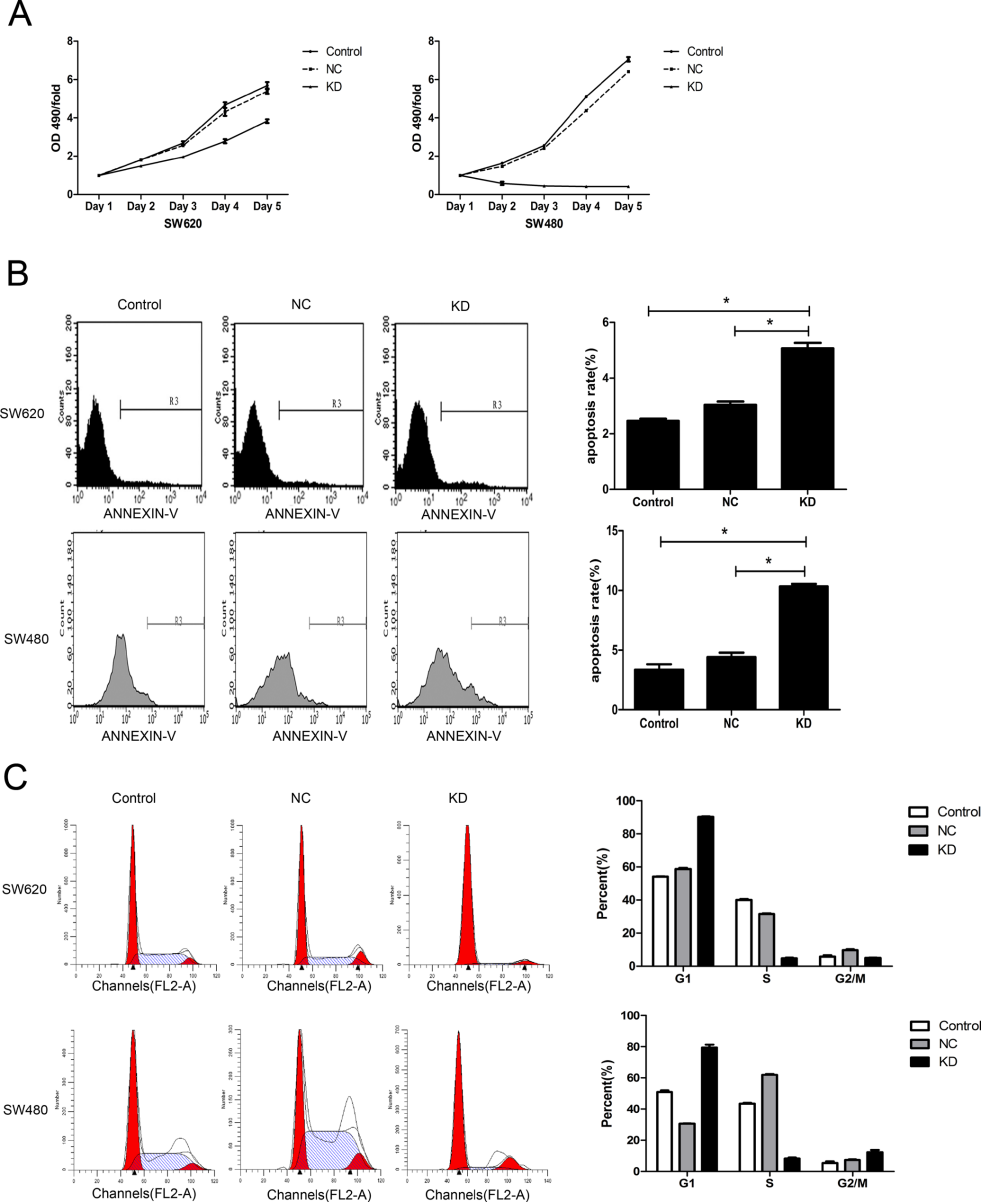
Effect of ZFX knockdown on cell proliferation, apoptosis and cell cycle in CRC cells (**A**) ZFX knockdown inhibited the proliferation of SW620 and SW480 cells (*p* < 0.05), as analyzed by MTT assay. (**B**) ZFX knockdown increased the incidence of apoptosis among SW620 and SW480 cells (*p* < 0.05). (**C**) ZFX knockdown arrested SW620 and SW480 cells in G1 phase (*p* < 0.05). C, no infection; NC, negative control shRNA; and KD, ZFX shRNA (KD3).

The effect of ZFX knockdown on the proliferative ability of CRC cells was assessed using MTT assays. The proliferation rates of SW620 and SW480 cells were dramatically reduced following ZFX knockdown (Figure 3A). In addition, flow cytometric analysis showed that ZFX knockdown significantly increased the incidence of apoptosis among the cells [SW620: NC vs. KD, *P* = 0.0001; SW480: NC vs. KD, *P* < 0.0001] (Figure 3B). Furthermore, ZFX knockdown also arrested cell cycle events. As shown in Figure 3C, ZFX knockdown significantly increased the G1 phase fraction in both cell types (SW620: NC vs. KD, *P* < 0.0001; SW480: NC vs. KD, *P* < 0.0001) while decreasing the S phase fraction (SW620: NC vs. KD, *P* < 0.0001; SW480: NC vs. KD, *P* < 0.0001). On the other hand, whereas the G2/M phase fraction was decreased the in SW620 cells (NC vs. KD, *P* = 0.0003), it was increased in SW480 cells (NC vs. KD, *P* = 0.0048). Taken together, these results indicate that ZFX knockdown leads to cell cycle arrest in both SW620 and SW480 cells.

### Effect of ZFX knockdown on CRC cell colony formation and sensitivity to 5-Fu

**Figure 4:**
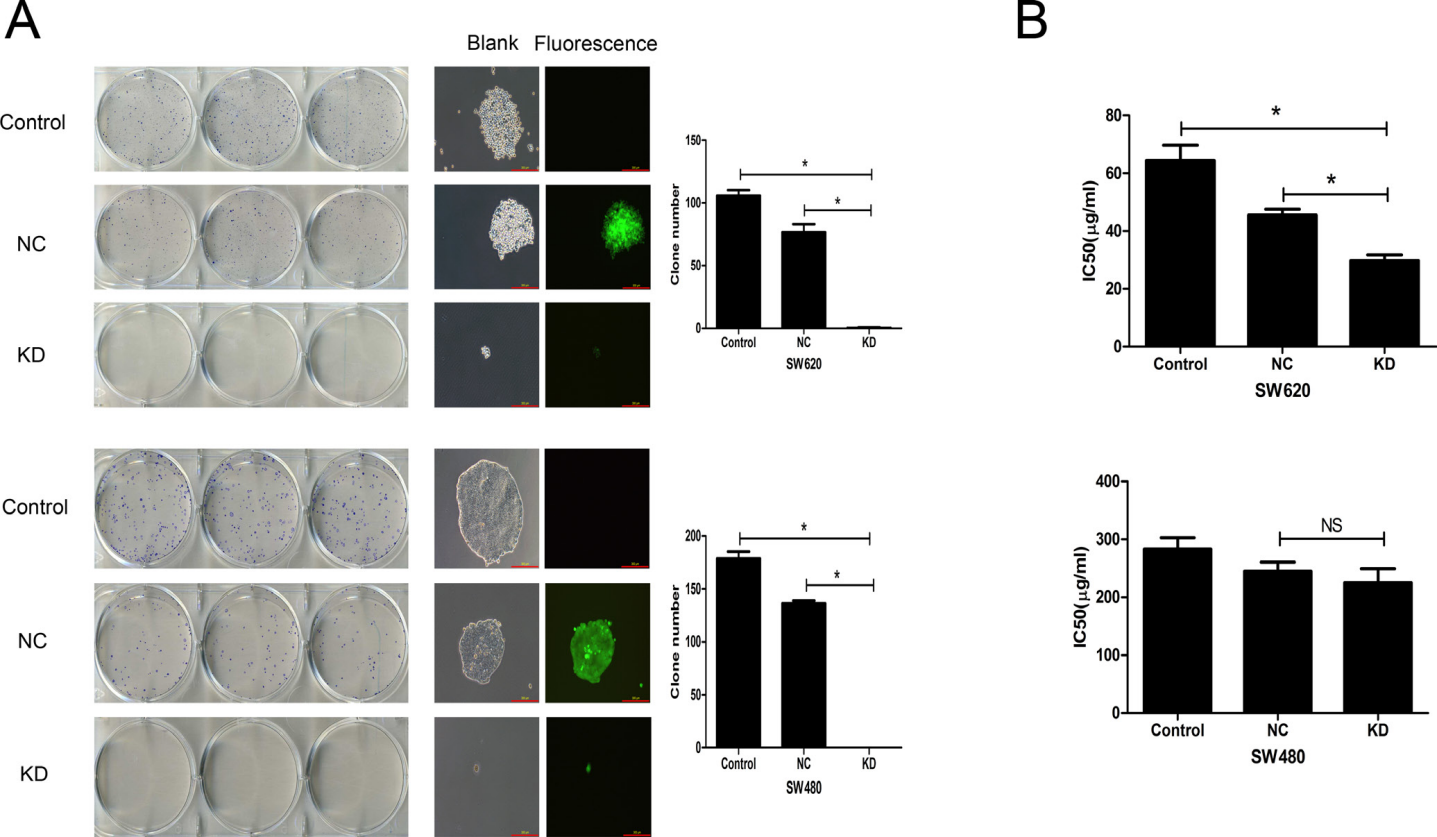
Effect of ZFX knockdown on CRC cell colony formation and sensitivity to 5-Fu (**A**) ZFX knockdown reduced the number and size of SW620 (top row) and SW480 cell (bottom row) colonies (*p* < 0.05). The left panel shows GIEMSA stained colonies in six well plates; the middle panel shows fluorescent images that capture the size of the colonies (magnification, 100×; scale bar, 300 μm); and right panel shows the colony numbers under each condition. (**B**) ZFX knockdown reduced the IC50 for 5-Fu in SW620 cells (*p* < 0.05) (left panel) but not in SW480 cells (*p* > 0.05) (right panel). Control, no infection; NC, negative control shRNA; and KD, ZFX shRNA (KD3).

To analyze the tumorigenic potential of ZFX in CRC, colony formation assays were performed. As shown in Figure 4A, a significant reduction in the number and size of SW620 and SW480 cell colonies was observed after ZFX knockdown (all *P* < 0.001). In addition, the effect of ZFX knockdown on drug sensitivity was analyzed by measuring the cytotoxicity of a chemotherapeutic drug, 5-Fu, in both SW620 and SW480 cells with or without ZFX expression. Cytotoxicity assays revealed that ZFX knockdown significantly reduced the IC50 for 5-Fu in SW620 cells (NC vs. KD: 45.6 ± 2.0 μg/ml vs. 29.7 ± 2.1 μg/ml, *p* = 0.0007) (Figure 4B), though no such effect was observed in SW480 cells (NC vs. KD: 244.8 ± 16.0 μg/ml vs. 225.1 ± 24.1 μg/ml, *p* = 0.302). This suggests ZFX may contribute to therapeutic resistance to 5-Fu in SW620 cells.

### Effect of ZFX knockdown on growth of CRC xenografts

**Figure 5:**
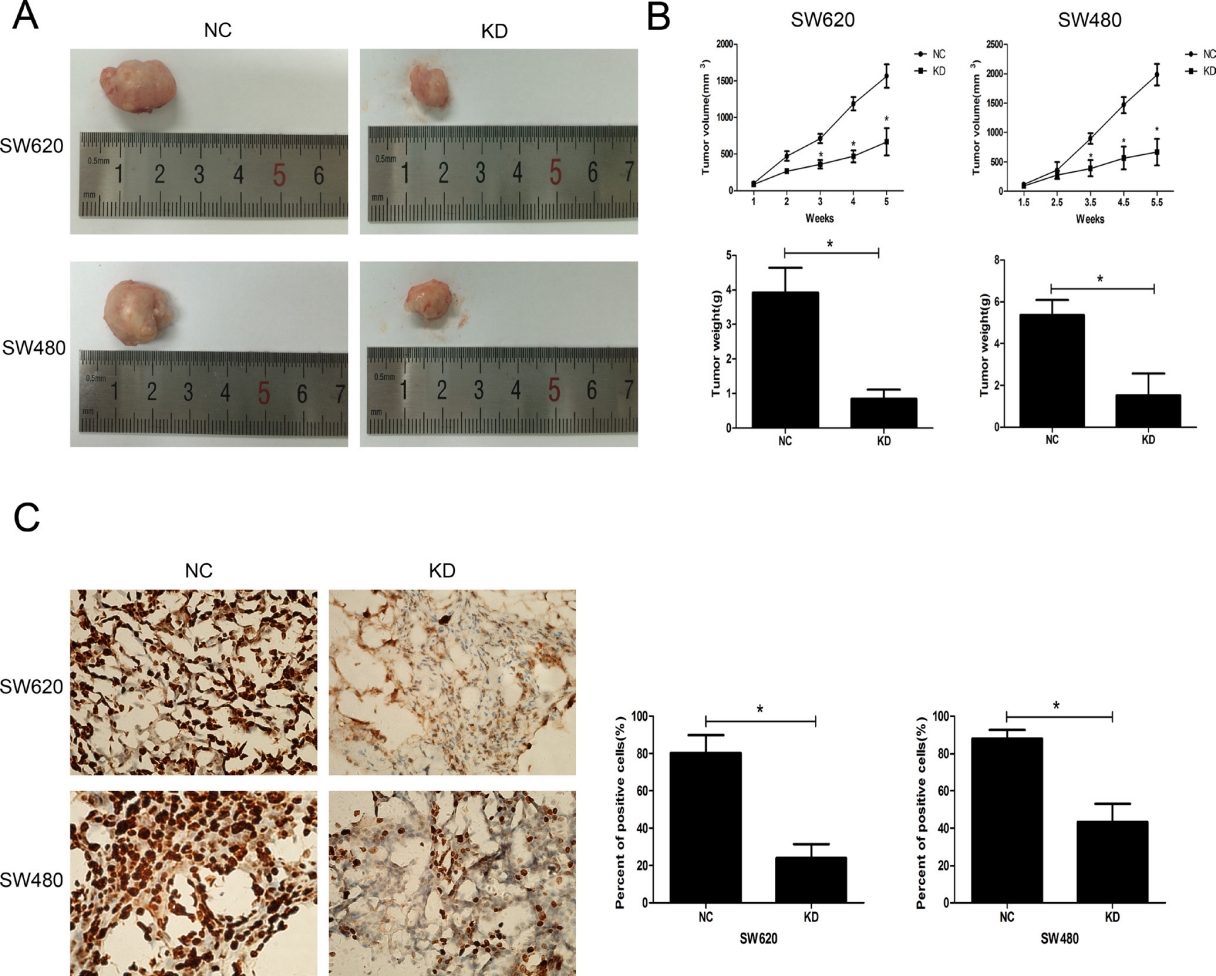
Effect of ZFX knockdown on the growth of CRC cell xenografts (**A**) Representative images of harvested tumors from nude mice subcutaneously injected with NC or ZFX KD SW620 and SW480 cells. Tumor sizes were decreased after ZFX knockdown. (**B**) Tumor volumes (upper panel) and weights (lower panel) were decreased after ZFX knockdown (*p* < 0.05). (**C**) Representative immunostaining of the Ki-67 proliferation marker in NC and ZFX KD tumors (left panel, 400× magnification). Numbers of Ki-67 positive cells were reduced in ZFX KD tumors (right panel) (*p* < 0.05).

The effect of ZFX on *in vivo* tumorigenicity was investigated using a xenograft model in which nude mice were subcutaneously injected with SW620 and SW480 cells transfected with NC or ZFX shRNA. As shown in Figure 5A, for both CRC cell types, the tumors harvested from the KD group were much smaller than those from the NC group. Both the volumes and weights of tumors from the KD group were significantly smaller than those from the NC group (*P* < 0.001) (Figure 5B). Furthermore, the proliferative ability of cells in the KD group was confirmed by immunohistochemical staining of a Ki67 marker. The staining (Figure 5C) suggested the Ki67-positive nuclear signal was significantly weaker in CRC cells in the KD group than in the NC group (*P* < 0.001).

### Microarray analysis of ZFX knockdown CRC cells

**Figure 6:**
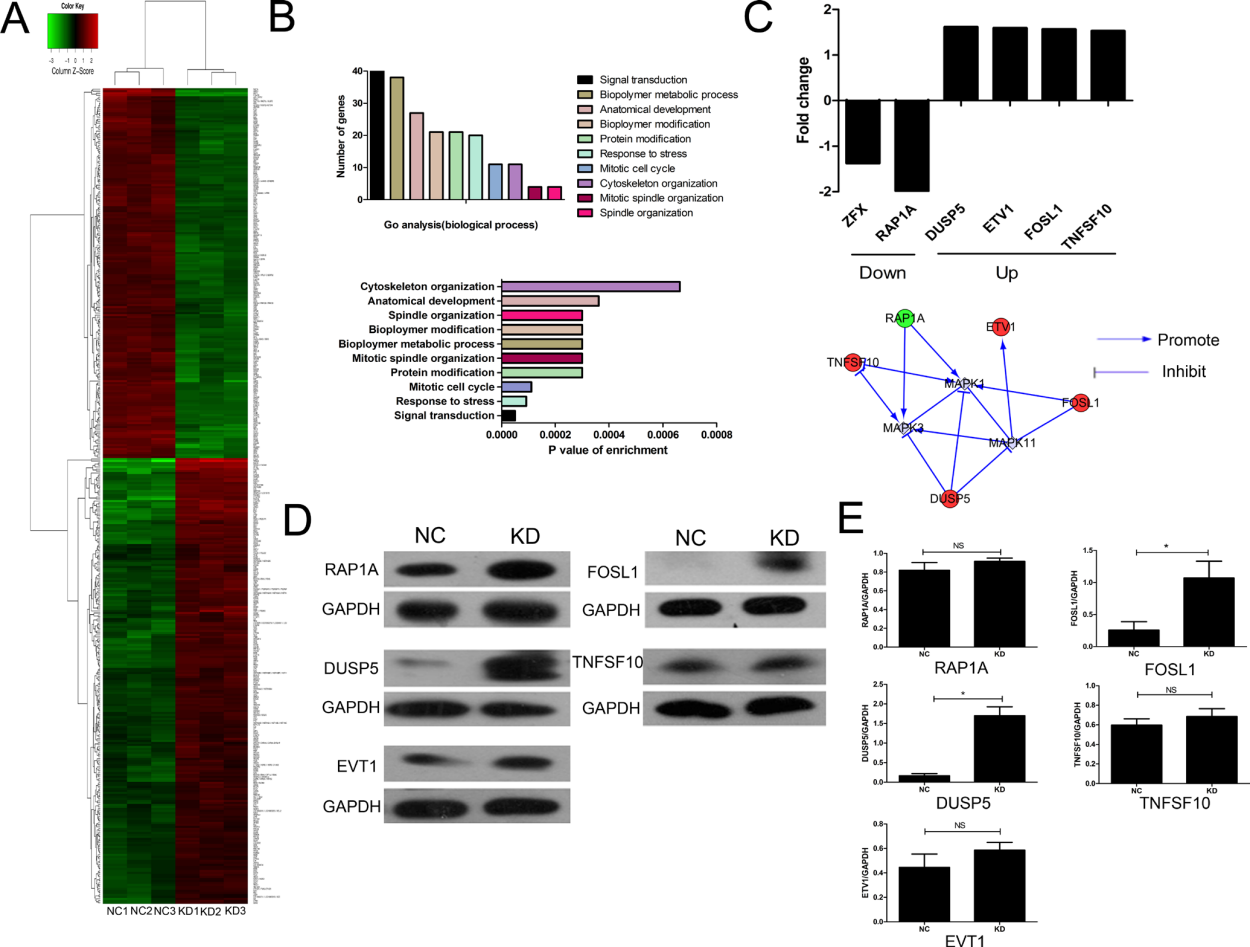
Microarray analysis and confirmation of the identified target proteins (**A**) Heat map depicting significantly affected genes (red color denotes upregulation and green denotes downregulation) in SW620 cells transfected with NC or ZFX shRNA. Each sample was processed in triplicate. (**B**) Gene ontology-based classification of gene numbers, expression, and significance probability. (**C**) Fold changes of five putative MAPK pathway-related genes (Upper). Knowledge-based interaction network for five selected targets in the MAPK pathway, constructed using the Reactome database (Lower). (**D**–**E**) Western blot confirmation (left panels) of RAP1A, DUSP5, EVT1, FOSL1 and TNFSF10 as targeted proteins (*p* < 0.05) and quantitation and normalization of their expression. GAPDH served as a reference control (right panel).

To explore the potential molecular mechanism of the ZFX contribution to the malignant characteristics of CRC cells, microarray analysis was performed using SW620 cells transfected with ZFX and NC shRNA. Based on the inclusion criteria, a total of 290 up-regulated genes and 196 down-regulated genes were identified following ZFX knockdown. The associated heat-map (Figure 6A) provides an overview of the significantly affected genes. GO enrichment analysis, categorizing the regulated genes into different sets of biological processes (Figure 6B), suggested that among the analyzed gene sets, genes related to signal transduction, stress response and mitotic cell cycle pathways were the top three showing the greatest changes in expression, based on significance probability. To identify specific potential targets of ZFX, the putative regulatory network based on the Reactome database was searched. However, due to the limited number of studies pertaining to ZFX, no direct intractable targets were identified in the Reactome database (data not shown). In a recent gastric cancer study, ZFX was shown to be closely linked with the MAPK signaling pathway, which contributes to CRC development [12]. We therefore focused our attention to selected genes that might be linked with the regulation of MAPK signaling pathway. Based on this bioinformatics analysis, fie genes related to MAPK signaling [RAP1A, Dual specifiity phosphatase 5 (DUSP5), Ets variant 1 (ETV1), FOS-like antigen 1 (FOSL1), and Tumor necrosis factor superfamily member 10 (TNFSF10)] showing 1.5 fold changes were selected from the microarray data (upper panel, Figure 6C). An interaction network was then established using the Reactome database by automatically adding key pathway molecules (MAPK1, MAPK3, and MAPK11) to the downstream of the five selected targets (lower panel, Figure 6C). To further validate these five targets, western blot analysis was performed and the results suggested that the expression of DUSP5 and FOSL1 were significantly upregulated after ZFX knockdown in SW620 cells (all *P* < 0.05) (Figure 6D–6E).

### Validation of DUSP5 gene as a ZFX target

**Figure 7:**
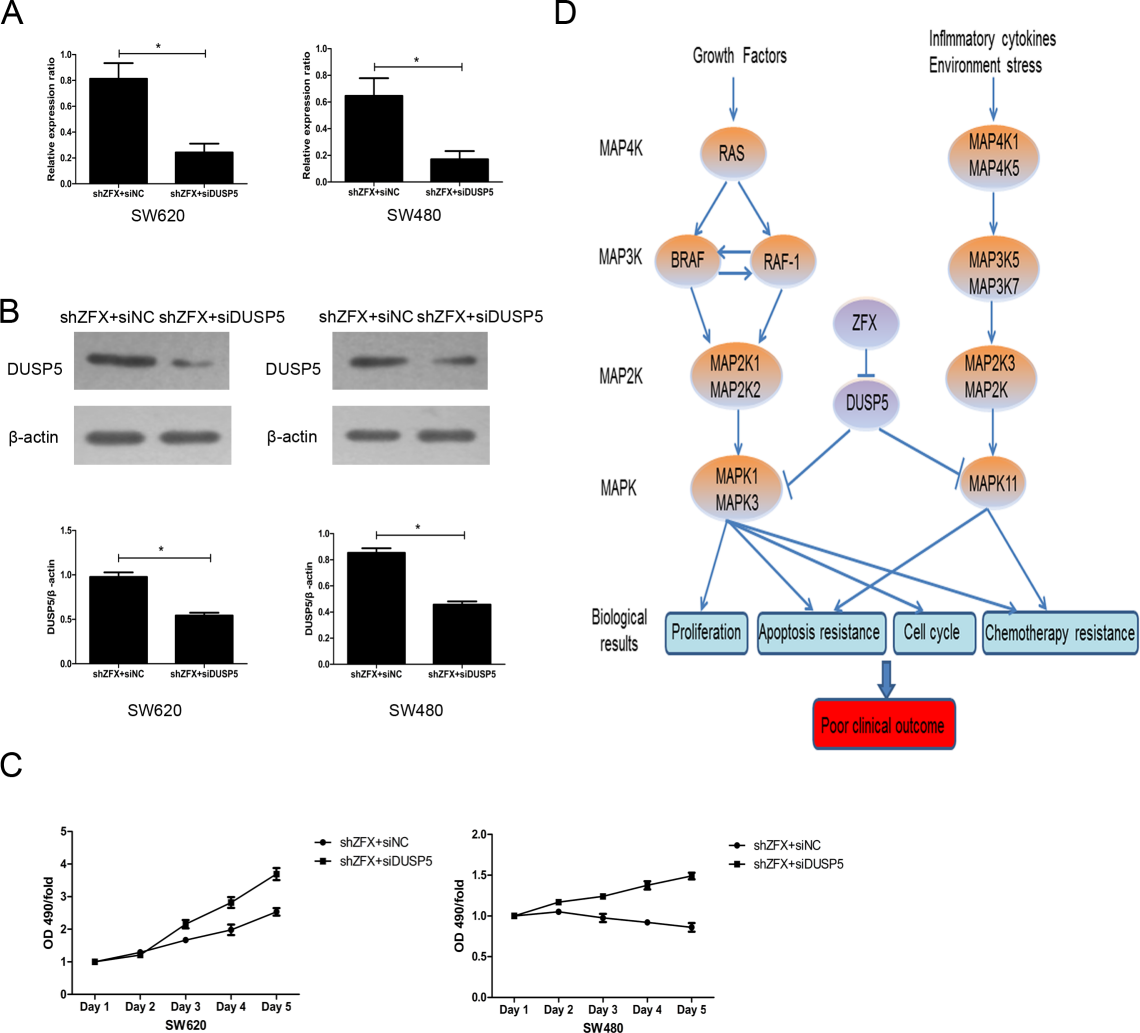
Validation of DUSP5 gene as a ZFX target (**A**) RT-PCR confirmed the mRNA expression of DUSP5 in ZFX-KD cells after siRNA transfection (*p* < 0.05). (**B**) Western blot confirming the protein expression of DUSP5 in ZFX-KD cells after siRNA transfection (*p* < 0.05). (**C**) DUSP5 knockdown rescued ZFX-mediated cell proliferation in ZFX-KD SW620 and SW480 cells. (**D**) Putative working model depicting the tentative mechanism by which ZFX promotes malignant progression and poor prognosis.

The information in the Reactome and Oncomine databases suggest FOSL1 likely acts as an oncogene that activates the MAPK pathway (data not shown). However, That is not consistent with our finding that ZFX knockdown resulted in upregulation of FOSl1 gene. We therefore proposed that DUSP5 is the dominant target of ZFX in SW620 and SW480 cells. To determine whether DUSP5 downregulation could rescue the growth-suppressive effects of ZFX knockdown, we used siRNA to ablate DUSP5 in ZFX-KD SW620 and SW480 cells (Figure 7A–7B). Then using MTT assays we showed that DUSP5 downregulation rescued the ZFX-mediated suppression in cell proliferation (Figure 7C). Based on these results and our microarray analysis, we proposed a putative working model depicting the tentative mechanism by which ZFX stimulates CRC malignant progression leading to a poor prognosis (Figure 7D). It appears that ZFX expression downregulates DUSP5, which sustains RAS- or MAP4K1/5-induced activation of the MAPK pathway involving MAPK1, MAPK3 and MAPK11. This ultimately results in the induction of oncogenic signaling with unfavorable clinical outcomes.

## DISCUSSION

In this study, we detected high ZFX expression in a majority of stage II/III CRC tissue samples tested, which correlated with tumor differentiation and stage along with its involvement in the CRC development. This observation is consistent with the recent finding by Yin et al. who reported that ZFX expression is aberrantly high in tongue squamous cell carcinoma tissues and is significantly correlated with tumor grade and stage [16]. Similarly, high ZFX expression is frequently detected in primary breast cancer tissues with metastatic potential, implying a role in tumor progression [9]. Our analysis based on the Kaplan Meier model predicted that stage II/III CRC patients with high ZFX expression would exhibit lower OS and DFS than those with lower ZFX expression. This observation is consistent with several recent studies suggesting ZFX expression is prognostic in nasopharyngeal and gallbladder carcinomas [17–18]. However, our subgroup analysis also confirmed that ZFX expression is prognostic in stage II and III patients who received 5-Fu-based chemotherapy. This is important, as recent prognostic assessments regarding chemotherapy in stage II/III CRC patients are controversial and a challenging problem for oncologists [19–20]. Although it has been proposed that other patient-related factors, such as lymphocyte count, could serve as prognostic markers, many of those have clinical limitations and a tendency to be influenced by uncertainties such as the overall condition of the patient [21]. In this regard, our findings not only demonstrate that ZFX expression may distinguish high-risk from low-risk patients within a single stage category, they suggest it could be a useful predictor of the efficacy of 5-Fu-based chemotherapy in those patients. These clinical findings are supported by our cytotoxicity assay, in which ZFX knockdown led to enhanced sensitivity to 5-Fu in SW620 cells, as well as by studies in which ZFX appeared to act as a crucial mediator of chemotherapy resistance to 5-Fu or cisplatin in cases of gastric and hepatocellular carcinoma [11, 13]. Through multivariate analysis, we identified ZFX expression and tumor stage as independent prognostic factors in stage II/III patients, which strengthens our conclusion that detection of ZFX expression may be useful for the current TNM staging system, which would in turn help to guide clinical management toward improved outcomes. Our data also aligned well with a recent comprehensive analysis by Amini et al. who suggested that ZFX is a putative diagnostic/prognostic biomarker in colon, bladder, and prostate cancer [22].

Although the biological function of ZFX has been investigated in several human malignancies [23–25], to the best of our knowledge it is the first detailed investigation of ZFX in CRC cells. Our findings show that ZFX knockdown significantly inhibits cell proliferation and *in vitro* colony formation, enhances apoptosis, induces cell cycle arrest. In addition, our xenograft studies establish that ZFX knockdown impairs the growth of CRC cells *in vivo*. These observations support the hypothesis that ZFX acts as an important positive regulator in CRC growth, which is in agreement with recent studies of the role of ZFX in renal and gastric cancers [10, 12].

To further elucidate the oncogenic mechanism by which ZFX promotes CRC progression, we performed a microarray analysis. We found that the genes affected by ZFX knockdown were significantly linked with signal transduction, stress response, and mitotic cell cycle. This suggests ZFX is involved in mediating oncogenic signaling that affects the response to chemotherapy and malignant proliferation. To identify a link between ZFX and a specific oncogenic pathway, we focused our attention on the MAPK pathway. This was in part because activation of the MAPK pathway was recently shown to contribute to oncogenicity in other cancers [12, 26], and also because this pathway plays a crucial role in many of the malignant characteristics of CRC, including uncontrolled proliferation and chemotherapy resistance [27, 28]. Based on a bioinformatics analysis, five MAPK pathway-related genes were identified from the ZFX knockdown microarray data and verified by western blot analysis. Ultimately, we proposed that DUSP5 is the primary target of ZFX. As a recently established negative regulator of the MAPK pathway [29], it was suggested that DUSP5 exerts anticancer effects by dephosphorylating extracellular regulated protein kinases in human malignancies [30–31]. Moreover, low Dusp5 expression was shown to be an adverse prognostic factor in patients with gastric/prostate cancer [31–32]. We therefore hypothesized that ZFX promotes CRC progression by downregulating DUSP5. To test this hypothesis, we employed siRNA techniques to knock down DUSP5 expression in ZFX-KD cells and observed that DUSP5 knockdown rescued ZFX-mediated cell proliferation in ZFX-KD cells. Based on this observation and microarray analysis, we suggest that upregulation of Dusp5 expression following ZFX knockdown attenuates MAPK signaling, which in turn inhibits growth *in vitro* and *in vivo*. However, further investigation is needed to confirm ZFX/DUSP5 signaling axis in the MAPK pathway.

In sum, our study not only suggests ZFX is a potentially useful predictor that could improve current methods for prognostic assessment of stage II/III CRC patients, but also that targeting ZFX is be a promising therapeutic approach in CRC patients.

## MATERIALS AND METHODS

### Patient data

A total of 290 paired samples CRC and adjacent normal tissue were collected from patients who underwent radical CRC surgery between January 2006 and December 2008 at the Sixth People's Hospital and Renji Hospital. Both of these hospitals are affiliated with Shanghai Jiao Tong University. The basic clinical features of the enrolled patients are presented in Table 1. Each of the CRC tissue samples was pathologically confirmed and staged according to the guidelines of the Union for International Cancer Control tumor-node-metastasis (TNM) staging system (7th edition). The following inclusion criteria were used to select the patients’ samples: 1) pathologically diagnosed with stage II or III CRC; 2) had no preoperative chemotherapy or radiotherapy; and 3) had complete hospital and follow-up records for the patient. Follow-up procedures were performed for enrolled patients every three to six months post-surgery, with regular laboratory and radiological examinations. OS was calculated as the time from the date of surgery to the date of death (resulting from any cause), or the date of the last follow-up. DFS was calculated as the time from the date of surgery to the date of the first recurrence or lymph node/distance metastasis. For post-operative chemotherapy, high-risk stage II patients (*n* = 121) and stage III patients (*n* = 124) received the standard therapeutic scheme for FOLFOX (5-Fu + Oxaliplatin + leucovorin). Written informed consent was obtained from each patient, and the study was conducted with the approval of the ethics committee of the both hospitals.

### Immunohistochemistry and staining evaluation

For details, please see Supplementary Materials.

### Cell culture and lentiviruses infection

For details, please see Supplementary Materials.

### Quantitative real-time reverse transcription PCR (QRT-PCR)

For details, please see Supplementary Materials.

### Western blot

For details, please see Supplementary Materials.

### MTT, apoptosis, cell cycle, colony formation and cytotoxicity assay

For details, please see Supplementary Materials.

### Xenograft models

For details, please see Supplementary Materials.

### Microarray analysis

Microarray analysis was performed using the GeneChip^®^ PrimeView^™^ Human Gene Expression Array (Affymetrix, USA). Briefly, total RNA was extracted in triplicate from SW620 CRC cells transfected with shRNA and the negative control. The quality of the RNA samples was assessed using Nanodrop 2000 (Thremo Fisher Scientific, USA) and 2100 Bioanalyzer (Aglient, USA). The 100 ng RNA samples were mixed with a poly-A RNA control and processed into double-stranded cDNA. *In vitro* transcription (IVT) of cRNA was then performed by adding 30 μl of IVT Master Mix (4 μl of 3′ IVT Biotin Label, 20 μl of 3′ IVT Buffer, 6 μl of 3′ IVT Enzyme, Affymetrix) to 30 μl of double-stranded cRNA. The generated cRNA was purified, quantified and labeled. Finally, arrays were hybridized in a GeneChip Hybridization Oven 645 (Affymetrix), washed in the GeneChip Fluidics Station 450 (Affymetrix) using GeneChip Hybridization Wash and Stain Kit (Affymetrix) and scanned using a Genechip Array scanner 3000 7G (Affymetrix).

Array data were normalized using log scale robust multi-array analysis (RMA) and were analyzed by R-Project software. Gene expression was deemed significant the fold change (FC) value was > 1.5 and *P* < 0.05. Gene Ontology (GO) was used to perform functional enrichment analysis, which included analyses of biological processes, cellular components, and molecular function. For statistical analysis of GO, Gene set enrichment analysis (GESA) and Fisher exact analysis were performed. As part of this study, potential downstream genes involved in the biological processes were selected for verification, and gene interaction networks were constructed using the Reactome database [15].

### Statistical analysis

Data are presented as the mean ± standard deviation (SD). Statistical analyses were performed using SPSS 17.0 statistical software (SPSS, USA). The correlations between ZFX expression and clinicopathological parameters were assessed using the χ^2^ test. Survival curves were constructed using the Kaplan Meier method, and statistical differences between the survival rates of subgroups was assessed using the log-rank test. Univariate and multivariate analyses based on the Cox proportional hazards regression model were carried out to identify significant prognostic indicators affecting OS among patients. For functional *in vitro* and *in vivo* assays, statistical significance between two groups was determined using a two-sided Student's *t* test. For all analysis, values of *P* < 0.05 were considered significant.

## SUPPLEMENTARY MATERIALS


