# Supplementary material for: High expression of Zinc-finger protein X-linked promotes tumor growth and predicts a poor outcome for stage II/III colorectal cancer patients
Source: Oncotarget. 2016 Feb 21;7(15):19680–92. doi: 10.18632/oncotarget.7547 (PMC4991411; doi:10.18632/oncotarget.7547)
Supplement: Supplementary file 1 [file oncotarget-07-19680-s001.pdf]

High expression of Zinc-finger protein X-linked promotes tumor growth and predicts a poor outcome for stage II/III colorectal cancer patients

Supplementary Materials

MATERIALS AND METHODS

Immunohistochemistry and staining evaluation

Paraffin-embedded CRC and the associated matched normal tissue samples were cut into 4 µm-sections. Each section was then dewaxed in xylene and rehydrated in alcohol gradient. After antigen retrieval under pressure for 15 min, the sections were immersed in 0.3% hydrogen peroxide solution to block endogenous peroxidase activity. Following three washes with PBS, the sections were incubated at 4°C overnight with primary antibody against ZFX (1:150, Santa Cruz Biotechnology, USA). The sections were then incubated at 37°C for 20 min with polyclonal goat anti-rabbit IgG (1:250, Abcam, UK). Diaminobenzidine reagent (Invitrogen Life Technologies, USA) was used to visualize the stain according to the manufacturer's instructions. Finally, the sections were counterstained with hematoxylin, dehydrated and sealed. Sections that were incubated with PBS instead of primary antibody were used as negative controls. For tumor tissues harvested from mice, a similar experimental procedure was used. These tissues were stained with Ki67 (1:200, Abcam, UK) primary antibody.

Staining evaluation was performed by two independent researchers. Both researchers were blinded to the clinicopathological parameters and the patients' outcome. An experienced pathologist evaluated any discrepant cases. In order to score the stains, five random fields of each section were selected. The scoring system was based on the immunoreactive score (IRS), which was defined based upon the product of Percentage of Positive cells (PP) and Staining Intensity (SI). PP was scored from 0 to 4 as follows: 0 (< 5%), 1 (5–25 %), 2 (> 25%–50%), 3 (> 50%–75%), and 4 (> 75%). SI was scored from 0 to 3 as follows: 0 (negative), 1 (weak), 2 (moderate), and 3 (strong). For statistical analysis, the final results were categorized as follows: low expression (IRS < 3) and high expression (IRS ≥ 3).

Cell culture and RNA interference

Each human CRC cell line (HCT116, SW480, Caco-2, LoVo, HT-29 and SW620) that was used as part of this study was purchased from the Type Culture Collection of the Chinese Academy of Sciences (Shanghai, China). The HCT116 and HT-29 cells were maintained in McCoy's 5A culture medium (Sigma-Aldrich, USA), while the SW480 and SW620 cells were maintained in L-15 culture medium (Gibco, USA). The LoVo and Caco-2 cells were maintained in F12K (Sigma-Aldrich, USA) and MEM (Gibco, USA) culture medium, respectively. All of the cell lines were incubated in a 5% CO2 atmosphere at 37°C.

In order to facilitate stable knock down of endogenous ZFX in CRC cells, three Short hairpin RNAs (shRNA) were designed using the following sequences: 5′-GCCTGAGAATGATCATGGA-3′ (KD1), 5′-CGGAAATTGATCCTTGTAA-3′ (KD2) and 5′-AAT CAGTCTCATTCACATA-3′ (KD3). The negative control shRNA sequence was: 5′-TTCTCCGAACGTGTCAC GT-3′. The synthesized DNA oligonucleotides containing these sequences were then annealed and inserted into the GV118 vector with GFP (Genechem, China). Verification of the inserted sequences was performed using DNA sequencing. To facilitate lentivirus manipulations, the GV118 vector, together with pHelper 1.0 and pHelper 2.0 plasmids (Genechem, China), were transfected into 293 T cells. Forty-eight hours after transfection initiation, the cultured supernatant containing the lentiviral particles was collected and purified. For lentivirus infection, CRC cells that were experiencing logarithmic growth were infected with lentivirus according to the multiplicity of infection (MOI). The lentivirus carried a copy of the Green Fluorescent Protein (GFP) and infection efficiency was assessed based on the numbers of GFP-expressing cells using florescence microscopy. Moreover, commercially generated small interference RNA (siRNA, GenePharma, China) was used to reduce DUSP5 expression in cells and its sequence was designed as follows: 5′-AAGCCAUGACCACAUGGACGA-3′. The sequence of designed negative control was as follows: 5′-UUCUUCGAACGUG UCACGUTT-3′. All the transfection processes were performed using Lipofectamine 2000 (Invitrogen Life Technologies) according to the manufacturer's instructions.

Quantitative real-time reverse transcription PCR (QRT-PCR)

Total RNA was isolated from cultured cells using Trizol reagent (Invitrogen Life Technologies) according to the manufacturer's instructions. The isolated RNA was

then reverse transcribed into cDNA using M-MLV reverse transcriptase (Promega, USA). The reaction was carried out using SYBR Premix Ex Taq (Takara, Japan) using the Thermal Cycler Dice Real Time System (Takara). The reaction conditions that were applied to facilitate the generation of cDNA were as follows: 1 cycle at 95°C for 30s, 45 cycles of a two-step PCR at 95°C for 5s and 60°C for 30s. The following primer sequences were used: ZFX-forward, 5′-GGCAGTCCACAGCAAG AAC-3′; ZFX-reverse, 5′-TTGGTATCCGAGAAA GTCA GAAG-3′; DUSP5-forward, 5′-GCAAGGTCCTGGTCCA CTGT-3′; DUSP5-reverse, 5′-AGGCGGAACTGCTTGGT CTT-3′; GAPDH-forward, 5′-TGACTTCAACAGCGACA CCCA-3′; and GAPDH-reverse, 5′-CACCCTGTTGCT GTAGCCAAA-3′. The mRNA expression of gene was normalized using the housekeeping gene and calculated using the $2^{-\Delta\Delta T}$ method. Each experiment was independently performed in triplicate.

Western blot

Total protein was isolated from the cultured cells using RIPA Lysis Buffer (Genechem). Protein samples were then separated on 10% SDS-PAGE and transferred to the polyvinylidene difloride membrane (Millipore, USA). The membranes were blocked for 1 hr at room temperature using TBST solution containing 5% skimmed milk. Subsequently, the membranes were incubated overnight at 4oC with the following primary antibodies: anti-ZFX (1:400, Santa Cruz Biotechnology, USA), RAP1A (1:3000, Abcam, UK), DUSP5 (5μg/ml, Abcam, UK), ETV1 (5 μg/ml, Abcam, UK), FOSL1 (1:5000, Abcam, UK), TNFSF10 (1:200, Abcam, UK) and β-actin (1:2000, Abmart, USA)/GAPDH (1:1000, Cell Signaling Technology, USA). The membranes were washed three times with TBST solution the following day and incubated with the respective secondary antibodies (1:5000, Santa Cruz Biotechnology, USA). Protein detection was performed using the Amersham ECL SelectTM detection system (GE Healthcare Life Sciences, USA) and signal was recorded using photographic film. The optical density of each protein was quantified using Quantity One software. β-actin and GAPDH expression were used as internal controls.

MTT assay

Both CRC cell groups were seeded into 96-well plates (2500/well for SW620 and 2000/well for SW480) in 200 μl of culture medium. After overnight incubation, 20 μl of MTT reagent (5 mg/L, Beijing Dingguo Changsheng Biotechnology, China) was added into each well. Following incubation at 37°C for 4 hrs, the culture medium was removed and 150 μl of DMSO reagent (Sinopharm Chemical Reagent, China) was added to allow complete dissolution of purple precipitates. After the plates were gently shaken for 10 min, the optical density (OD) of each well was determined using a microplate reader (Biotek, USA) at a wavelength of 490 nm. The absorbance values were used to determine the growth rates of cells, which was calculated as follows: (OD of the sample on the test day)/(OD of the sample at Day 1).

Apoptosis

The apoptosis of CRC cells was measured using the AnnexinV-APC single staining method. Briefly, the cells were washed with D-Hanks solution and were trypsinized. Thereafter, they were collected and centrifuged at 1500 rpm for 5 min and then washed with PBS. Next, after washing with AnnexinV 10 × binding buffer, the cells were incubated with 10 μl AnnexinV-APC reagent (eBioscience, USA) in the dark for 15 min at room temperature. Finally, the cell suspension was transferred to the 96-well plate (200 μl/well) and analyzed by flow cytometry (Millipore, USA).

Cell cycle assay

Each group of cells were washed with D-Hanks solution, digested with trypsin and collected by centrifugation at 1500 rpm for 5 min. After two washes with ice-cold PBS and fixation in 70% ethanol for 1 hr, cells were re-suspended in 1ml staining buffer containing PI (2 mg/ml, Sigma-Aldrich) and RNase A (10 mg/ml, Fermentas, Canada). Finally the cells were subjected to FACSCalibur flow cytometer (BD Biosciences) for cell cycle analysis.

Colony formation assay

Cells in the logarithmic phase were digested, re-suspended and seeded onto the 6-well plates (800/well). After incubation in a 5% CO2 atmosphere at 37°C for two weeks, the cells were washed with PBS and fixed with paraformaldehyde (1 ml/well) for 60 min. Following two washes with PBS, the cells were stained with GIEMSA reagent (500ml/well, Chemicon, Japan) for 20 min. Finally, the colonies were photographed and counted under a fluorescence microscope (Olympus, Japan).

Cytotoxicity assay

SW620 and SW480 cells were seeded onto the 96-well plate (2×104/well) and treated with gradient of 5-FU drug (0.5μg/ml to 1000μg/ml) for 48 hrs. Then cell viability was assessed using the CCK-8 method (10μl/well, Dojindo, Japan). After incubation for 2 hrs, cells were subjected to a microplate reader for recording an absorbance at a wavelength of 450 nm. The 5-FU sensitivity was evaluated based on IC50, which was defined as the concentration inducing 50% inhibition of cell viability.

Xenograft models

A total of 16 athymic nude male BALB/c mice (4–5 weeks old, 15–18g) were purchased from Shanghai Labarotary, Animal Research Center. The experimental

protocol was approved by the Animal Care and Use Committee and the Ethics Committee of the Sixth People's Hospital Affiliated to Shanghai Jiao Tong University. Both type of CRC cells (5.0 × 106 each) in the logarithmic phase were suspended in 200 μL of serum-free medium and then injected subcutaneously into the left hind flank of each mouse. Tumor volumes were monitored every week using a caliper and calculated according to the following formula: volume = ab2/2, where "a" and "b" was defined as length and width of the tumor respectively. Four weeks later, mice were sacrificed, tumors were harvested and tumor weights were measured before sending for immunohistochemical analysis.
